# Food Allergy and *Helicobacter pylori* Infection: A Systematic Review

**DOI:** 10.3389/fmicb.2016.00368

**Published:** 2016-03-23

**Authors:** Zheng Feei Ma, Noorizan A. Majid, Yoshio Yamaoka, Yeong Yeh Lee

**Affiliations:** ^1^Department of Human Nutrition, University of OtagoDunedin, New Zealand; ^2^School of Medical Sciences, Universiti Sains Malaysia Kubang Kerian, Malaysia; ^3^Department of Medicine, Gastroenterology and Hepatology Section, Baylor College of Medicine, HoustonTX, USA; ^4^Department of Environmental and Preventive Medicine, Oita University Faculty of MedicineYufu, Japan

**Keywords:** *Helicobacter pylori*, atopic disease, food allergy, allergy, food sensitivity

## Abstract

**Introduction:** Based on the hygiene hypothesis, a low prevalence of *Helicobacter pylori (H. pylori)* infection may explain the recent high prevalence of allergic diseases including food allergy. However, there are very few studies that investigate the relationship between *H. pylori* and food allergy.

**Summary:** We searched for PubMed, Ovid Medline and the Cochrane library for relevant articles published in English from inception to November 2015. The inverse relationship between *H. pylori* and food allergy remains unproven because of contradictory and limited evidence at the moment. Likewise, only limited studies have examined the relationship between CagA; one of *H. pylori* virulence factor and food allergy. On the other hand, *in vitro* evidence seems to point out a role of *H. pylori* in the causation of food allergy. The inconsistent results from epidemiological data may be due to small sample size, heterogeneous populations and unstandardised methods or food allergens.

**Conclusion:** Available studies do not support the role of *H. pylori* in food allergy.

## Introduction

*Helicobacter pylori* (*H. pylori*) is a major cause of various gastroduodenal diseases, including peptic ulcer disease and gastric cancer (Suerbaum and Michetti, 2002). Nearly half of the world population are infected with *H. pylori* (Peek and Blaser, 2002) with a higher prevalence in developing countries (∼80%; Suerbaum and Michetti, 2002), although it is recognized that some populations have unexpectedly low rates (Lee et al., 2013). Nevertheless, the overall prevalence of *H. pylori* infection has decreased in recent years because of effective eradication therapy and improvement in hygiene and living environment.

A low prevalence or loss of *H. pylori* is associated with a reduced risk for gastric cancer (Derakhshan and Lee, 2012; Lee and Derakhshan, 2013), however, the prevalence of allergic diseases may increase if the hygiene hypothesis holds true. The hygiene hypothesis proposed that an improved condition in early life reduces the exposure to various childhood infections, which might promote an increased in the prevalence of atopic disorders (Strachan, 1989). While an improved living condition in early life reduces exposure to various childhood infections, however, atopic disorders may increase and this is the basis of the hygiene hypothesis (Strachan, 1989). Only limited data are available to support the hygiene hypothesis in relation to food allergy.

Most studies focus on the role of *H. pylori* in allergic diseases such as asthma. However, there are only few studies that investigate the relationship between *H. pylori* and food allergy. This is despite that food allergy is as common as asthma or other allergic diseases if not more prevalent. The aim of this review was to highlight the increasing prevalence of food allergy in the West and Asia and the role of *H. pylori* to this increase.

## Search Strategy

We searched for PubMed, Ovid Medline and the Cochrane library for relevant articles published in English from inception to November 2015. Various combinations of the following terms were used: *Helicobacter pylori*, *H. pylori*, *H. pylori* infection, food allergy, allergic disease and atopic disease. We also located additional publications from reference lists of the retrieved studies. Relevant articles were selected for full texts after screening through the titles, abstracts and descriptors of the studies which met the eligibility of our review. We selected the studies for review if they investigated the relationship between *H. pylori* and food allergy in humans. Studies were excluded if they did not use *H. pylori* and food allergy as an outcome in the analysis.

## Epidemiology of Food Allergy

Food allergy is defined as an immunologically mediated process in response to a specific food response (Bruijnzeel-Koomen et al., 1995). Compared to previous epidemiological surveys (Gupta et al., 2007; Mullins, 2007; Branum and Lukacs, 2009), recent figures suggest that food allergy is more prevalent, affecting ∼6% of children and 3–4% of adults (Wang and Sampson, 2011). In the United States, the prevalence of food allergy in children aged 0–17 years had increased from 3.4% in 1997–1999 to 5.1% in 2009–2011 (Jackson et al., 2013).

## Relationship Between Food Allergy and *H. pylori* Infection

Only two studies by the same author reported a positive association between food allergy and *H. pylori* infection (**Table 1**). Corrado et al. (2000) reported that children with food allergy had a higher anti-*H. pylori* IgG concentration than those without food allergy (*p* < 0.05). An earlier study by, Corrado et al. (1998) reported that 30% of their patients with food allergy had a higher anti-*H. pylori* IgG concentration.

**Table 1:** A summary of studies which investigated the relationship between food allergy and *Helicobacter pylori* infection.

Reference	Type of population; Age (range); sample size	Underlying disease	*H. pylori* association	Type of food allergen
Baccioglu et al., 2008	Adults; 17–74; 90	Dyspeptic symptoms	Neutral	Food allergens supplied by Allergopharma (Reinbek, Germany)
Bartuzi et al., 2000	Adults; 16–60; 141	Group 1: atopic diathesis (i.e., dyspepsia and abdominal pains); group 2 (as controls): dyspepsia and abdominal pains related to chronic gastritis; group 3 (as controls): functional dyspepsia	Positive	Pork, veal, cocoa, fish, beer, apples, rice, hen’s meat, eggs, oranges, peas, onions, beans, strawberries, potatoes, celery, milk, peanuts, carrots, wheat flour, and tomatoes.
Corrado et al., 1998	Children; 5–12; 90	Group 1: no reported underlying diseases but only food allergic; group 2: atopic asthma; group 3: inflammatory bowel disease.	Positive	Casein, lactalbumin, β-lactoglobulin, ovalbumin, rice, soya, wheat, and fish
Corrado et al., 2000	Children; 4–12; 90	Group 1: food allergic and atopic dermatitis; group 2 (as controls) : food allergic and gastrointestinal symptoms; group 3 (as controls): atopic asthma	Positive	Casein, lactalbumin, β-lactoglobulin, ovalbumin, rice, soya, wheat, and fish
Figura et al., 1996	Adults; age not reported; 91	Group 1: symptomatic food allergy; group 2 (as controls): respiratory allergy and no known underlying disease.	Positive (only for *Cag-A*-positivity)	Alimentary or respiratory antigen by Pharmacia CAP System RAST FEIA (Uppsala, Sweden).
Figura et al., 1999	Children and adults; 6–60; 91	Group 1: symptomatic food allergy; group 2 (as controls): not specified	Positive (only for *Cag-A-*positivity)	Alimentary antigen by Pharmacia CAP System RAST FEIA
Konturek et al., 2008	Adults; 25–65; 62	No known underlying diseases	Negative	Not specified
Kolho et al., 2005	Children; 5–15; 74	Abdominal symptoms	Neutral	Wheat, fish, peanut, egg, soybean and cow’s milk


On the other hand, three studies report no association between food allergy and *H. pylori* infection (**Table 1**). Baccioglu et al. (2008) reported no significant difference in the prevalence of food allergy between patients with and without *H. pylori* infection. In addition, Figura et al. (1996) reported no difference between anti-*H. pylori* IgG levels between patients with food allergy and controls. Also, a study by Kolho et al. (2005) reported no difference in food specific IgE concentration between *H. pylori* positive and negative children. Only one study by Konturek et al. (2008) reported that patients with *H. pylori* infection had a reduced risk of food allergy. However, it is unknown if the reduced risk of food allergy was associated with the presence of other commensal bacteria (Cao et al., 2014).

It is difficult to interpret these contradictory results but many of these studies were limited by small sample sizes (ranged from 62 to 141) and heterogeneous methodologies (**Table 1**). For example, study by Corrado et al. (1998) enrolled food-allergic patients with unknown underlying disease, and his later study enrolled food-allergic patients with atopic disease and gastrointestinal symptoms (Corrado et al., 2000), raising the possibility for a type 1 error. Where else, Baccioglu et al. (2008) enrolled patients with dyspeptic symptom which might cause a type II error. The age range of patients was broad across studies: children aged 4–17 years and adults aged 18–74 years. One study reported their results of children and adults as a group (Figura et al., 1999). The types of food allergens were also different across studies (**Table 1**).

## How *H. pylori* Causes Food Allergy?

Food allergen is defined as those specific components of food or ingredients within food that are recognized by allergen-specific immune cells and elicit specific immunologic reaction, resulting in characteristic symptoms (Boyce et al., 2010). A T cell-mediated suppression or ‘oral tolerance’ will usually occur when the GI tract is exposed to food allergens (Faria and Weiner, 2005). However, this mechanism seems to fail in individuals with food allergy. The allergen causes inflammation of the GI mucosa, which subsequently increases the permeability to food antigens (Bock, 1980). Allergic sensitization then occurs and the resultant T-helper-2 (Th2) response leads to increase production of IgE, which then binds to the receptor on the mast cell surfaces located in the skin and the GI tract (Gould and Sutton, 2008).

The process of allergic sensitisation described above seems to be exacerbated by the presence of *H. pylori*. The passage of intact food proteins is increased across the gastric epithelial barriers if *H. pylori* is present (Matysiak-Budnik et al., 2004). In addition, gastric mucosa in patients with food allergy may be more hyperaemic and edematous (Ramsay et al., 2010), which further promote *H. pylori* adhesion. Previous studies have reported a relationship between an increase in food antigens’ absorption by the gut and the development of food allergy in those harboring this bacterium. *H. pylori* also causes inflammation of the GI mucosa and this results in a greater mucosal permeability which then allows more transition of food allergens (Borch et al., 1998; Fukuda et al., 2001). The food allergens bind to IgE on the mucosal mast cells, which then induce IgE-mediated histamine release from the human basophils *in vitro* (Aceti et al., 1991). The evidence indicates that infection of *H. pylori* may exacerbate the severity of IgE-mediated food allergy.

## Role of Caga Virulence Factor in Food Allergy

Colonization of *H. pylori* in the gastric mucosa stimulates release of cytokines such as interleukin (IL)-8 (Takahashi et al., 1998). IL-8 is one of the main mediators of inflammation of gastric mucosa during the course of *H. pylori* infection. IL-8 synthesis intensifies within the inflamed gastric epithelium (Huang et al., 1995) and cytotoxin-associated gene A (CagA) positivity was found to be an important stimulant for IL-8 (Leonard and Yoshimura, 1990). CagA is a 120–145 kda protein encoded on the Cag pathogenicity island (PAI; Hatakeyama and Higashi, 2005). There is a high prevalence of CagA-positive *H. pylori* in Asia, particularly in the Southeast Asian countries (93%; Sahara et al., 2012) and China (96%; Zhou et al., 2004).

There are studies which have investigated the relationship between food allergy and virulence factor of *H. pylori* (**Table 1**). CagA-positive patients could worsen food allergy as shown by Figura et al. (1996) because CagA stimulates the gastric cells to secrete high levels of inflammatory cytokines (Censini et al., 1996). For example, Figura et al. (1999) reported a higher IgE concentration was found in those with CagA-positive than those with CagA-negative patients. Another study by Figura et al. (1996) reported that CagA seropositivity was significantly higher in patients with food allergy than in controls (*P-*value = 0.03).

However, some studies (Corrado et al., 2000; Kolho et al., 2005) report no association between CagA positivity and food allergy (**Table 1**). For example, Kolho et al. (2005) reported no correlation between the food specific IgE and the severity of *H. pylori* infection as assessed by the presence of CagA antibodies.

There is no clear evidence to support that eradication of *H. pylori* worsens or improves food allergy reactions. (Figura et al., 1996, 1999; Corrado et al., 2000; Kolho et al., 2005). Very few studies have investigated on this research question. Even though there is a relationship between *H. pylor*i infection and food allergy, the available data do not prove causality.

## Food Allergy and *H. pylori* in Asia

Surveys using self-reported methodology seem to overestimate the prevalence of food allergy in the Asian populations. For example, Leung et al. (2009) reported that the prevalence rate of parent-reported, adverse food reaction in Hong Kong was 6.7%, and this was higher than doctor-diagnosed adverse food reaction (4.6%). However, some Asian countries use oral food challenges to diagnose food allergy in their populations (2013). But the methods used to diagnose food allergy are not standardized across Asian studies. Therefore, it is unclear whether the prevalence of food allergy in Asia reflects the true prevalence of food allergy in their populations.

Nevertheless, food allergy is shown to be on the increase in Asia. For example, Hu et al. (2010) reported that the prevalence of IgE-mediated food allergy has increased from 3.5 to 7.7% in children aged 0–2 years living in China between 1999 and 2009. However, many Asian countries do not have any data or have incomplete data on food allergy (Prescott et al., 2013). A meta-analysis by the National Institute of Allergy and Infectious Disease reported that the estimated prevalence of food allergy is generally between 1 and 10% from available Asian data, with a higher prevalence in children (Boyce et al., 2010). At the moment, there are no reported studies from Asia on the relationship between food allergy and *H. pylori*.

Recent data indicate a declining prevalence of *H. pylori* in Asia (Nakajima et al., 2010). This decline may explain the increasing prevalence of food allergy in Asia, if the hygiene hypothesis is true. However, it cannot be ruled out that other factors such as changing of food patterns to a more westernized diet (Prescott et al., 2013) and the interactions between gene and environmental factors (Kauffmann and Demenais, 2012) may also contribute to the increase in prevalence of food allergy in this region.

## Treatment Strategies

Currently, there is no cure for food allergy. Allergen avoidance and pharmacological treatments are used to manage food allergy (Sicherer and Sampson, 2014). Antihistamines and mast cell stabilizers are typically used over long term. Patients should be educated about dietary avoidance of causative food allergens in order to minimize the risk of food allergy reactions. At the same time, patients or their parents should be taught on the management of acute food allergic reactions based on the Guidelines for the Diagnosis and Management of Food Allergy in the United States (Boyce et al., 2010; **Table 2**).

**Table 2:** A summary of the guidelines for the diagnosis and management of food allergy in the United States (Boyce et al., 2010).

Dietary Management of Individuals with food allergy
Dietary avoidance of specific allergens in IgE and/or non-IgE-mediated food allergy
Nutritional counseling and regular monitoring of nutritional status especially for children who are growing
Education and training on how to identify the food allergens used as ingredients on the food labels
Follow-up testing on the specific food to which the patients are allergic to


## Conclusion

Available studies do not fully support an inverse relationship between *H. pylori* and food allergy. Furthermore, there are no studies that have been published to investigate the relationship since 2008. Although *in vitro* evidence points to a role of *H. pylori* in food allergy, the current data on human studies are less convincing. Therefore, further investigations are needed to better understand the role of *H. pylori* in food allergy which is an increasingly common condition especially among children both in the West and in Asia.

## Author Contributions

All authors have contributed substantially to the conception or design of the work, or the acquisition, analysis, or interpretation of data for the work: ZM wrote the first draft of the manuscript; NM, YY, and YL revised and provided feedbacks for the manuscript. All authors have drafted and revised the work critically for important intellectual contents. All authors approved the final version to be published. All authors agreed to be accountable for all aspects of the work in ensuring that questions related to the accuracy or integrity of any part of the work are appropriately investigated and resolved.

## Conflict of Interest Statement

The authors declare that the research was conducted in the absence of any commercial or financial relationships that could be construed as a potential conflict of interest.
