# Corrigendum: Food Allergy and *Helicobacter pylori* Infection: A Systematic Review

**DOI:** 10.3389/fmicb.2016.01232

**Published:** 2016-08-08

**Authors:** Zheng Feei Ma, Noorizan Abdul Majid, Yoshio Yamaoka, Yeong Yeh Lee

**Affiliations:** ^1^Department of Human Nutrition, University of OtagoDunedin, New Zealand; ^2^School of Medical Sciences, Universiti Sains MalaysiaKubang Kerian, Malaysia; ^3^Department of Medicine, Gastroenterology and Hepatology Section, Baylor College of MedicineHouston, TX, USA; ^4^Department of Environmental and Preventive Medicine, Oita University Faculty of MedicineYufu, Japan

**Keywords:** *Helicobacter pylori*, atopic disease, food allergy, allergy, food sensitivity

Reason for Corrigendum:

Due to an oversight, the first name of the first author was misspelled as Zheng Fei Ma instead of Zheng Feei Ma. The correct name of the first author should be Zheng Feei Ma and has been corrected. The authors apologize for the mistake. This error does not change the scientific conclusions of the article in any way.

The original article has been updated.

## Author contributions

All authors have contributed substantially to the conception or design of the work, or the acquisition, analysis or interpretation of data for the work: ZM wrote the first draft of the manuscript; NA, YY, and YL revised and provided feedbacks for the manuscript. All authors have drafted and revised the work critically for important intellectual contents. All authors approved the final version to be published. All authors agreed to be accountable for all aspects of the work in ensuring that questions related to the accuracy or integrity of any part of the work are appropriately investigated and resolved.

### Conflict of interest statement

The authors declare that the research was conducted in the absence of any commercial or financial relationships that could be construed as a potential conflict of interest.

